# Circulating cell-free DNA as a predictive marker for distant metastasis of hepatitis C virus-related hepatocellular carcinoma

**DOI:** 10.1038/sj.bjc.6604034

**Published:** 2007-10-16

**Authors:** Y Tokuhisa, N Iizuka, I Sakaida, T Moribe, N Fujita, T Miura, S Tamatsukuri, H Ishitsuka, K Uchida, S Terai, K Sakamoto, T Tamesa, M Oka

**Affiliations:** 1 Department of Surgery II, Yamaguchi University Graduate School of Medicine, 1-1-1 Minami-Kogushi, Ube, Yamaguchi 755-8505, Japan; 2 Department of Complementary Medicine, Yamaguchi University Graduate School of Medicine, 1-1-1 Minami-Kogushi, Ube, Yamaguchi 755-8505, Japan; 3 Department of Gastroenterology and Hepatology, Yamaguchi University Graduate School of Medicine, 1-1-1 Minami-Kogushi, Ube, Yamaguchi 755-8505, Japan; 4 Molecular Diagnostics R&D Department, Molecular Diagnostics Division, Roche Diagnostics K.K., 6-1, Shiba 2-chome, Minato-ku, Tokyo 105-0014, Japan

**Keywords:** HCV, HCC, blood testing, cell-free DNA, real-time PCR, *GSTP1*

## Abstract

In a previous study, we showed that levels of cell-free DNA (cfDNA) were significantly higher in sera of patients with hepatocellular carcinoma (HCC) associated with hepatitis C virus (HCV) than in sera of non-HCC patients with HCV. To confirm this finding, we analysed serum cfDNA levels in a cohort of 96 patients with HCV-related HCC and in 100 HCV carriers without known HCC. Again we found that serum cfDNA levels were significantly higher in HCC patients than in HCV carriers (115.9±98.3 *vs* 34.4±40.4 ng ml^−1^ (mean±s.d.), *P*<0.0001). Of 87 eligible patients who underwent curative hepatectomy, those with a high cfDNA level had a significantly shorter overall survival (OS) time than those in whom the cfDNA level was not high. Cox proportional hazards model showed the cfDNA level to be an independent prognostic factor for OS and cancer recurrence in distant organs. Our results suggest that the serum cfDNA level reflects the metastatic potential of HCV-related HCC and that it can be a useful predictive biomarker for distant metastasis after curative surgery.

Hepatocellular carcinoma is one of the most common cancers, with an estimated 564 000 new cases registered worldwide in 2000 (Parkin *et al*, 2001), and it represents a major international health problem because its incidence is increasing in many countries (Deuffic *et al*, 1998; Llovet *et al*, 2003; El Serag, 2004). Particularly in Europe and North America, the incidences of HCC have increased markedly in the last decade and will increase further in the upcoming two decades due to hepatitis C virus (HCV) infection (Deuffic *et al*, 1998; El Serag, 2004). Despite much effort in HCC research, the prognosis of HCC remains poor because of both a high frequency of intrahepatic recurrence (IHR; Iizuka *et al*, 2003, 2004; Llovet *et al*, 2003) and high mortality of associated with extrahepatic recurrence (EHR) in distant organs (Itoh *et al*, 2002; Yang *et al*, 2007). Therefore, new non-invasive prognostic markers are urgently needed to improve the prognosis of HCC, especially of HCV-related HCC.

It may become possible to use serum biomarkers to screen for HCC patients at high risk for recurrence (Mann *et al*, 2007). To date, many candidate markers have been reported in relation to the clinical outcome of HCC patients (Marrero and Lok, 2004; Kuramitsu and Nakamura, 2006). In addition to these biomarkers, circulating cell-free DNA (cfDNA) has attracted a great deal of attention as an easy-to-use tool for evaluation of the malignant potential of cancer (Gautschi *et al*, 2004; Umetani *et al*, 2006). By means of real-time polymerase chain reaction (PCR) assay, we found that cfDNA levels were significantly higher in sera from patients with HCV-related HCC than in sera from HCV carriers without known HCC (Iizuka *et al*, 2006a). An additional intriguing finding was that cfDNA levels increased in parallel with tumour dedifferentiation and were positively associated with tumour size. Ren *et al* (2006) showed that levels of circulating cfDNA correlated inversely with the prognosis of HCC, in most cases attributable to hepatitis B virus (HBV) infection, suggesting that cfDNA may be a robust predictive marker for the prognosis of HBV-related HCC. However, a marker specific to HBV-related HCC may not be useful for HCV-related HCC because there are many differences in genetic changes and the clinical course between the two types of HCC (Iizuka *et al*, 2002; Llovet *et al*, 2003). We were prompted to examine whether cfDNA levels were predictive of outcomes in a larger cohort of patients with HCV-related HCC. Our present study showed for the first time in a large study group that the cfDNA level in the bloodstream can function as a predictor for overall survival (OS) and EHR in distant organs after curative hepatectomy in patients with HCV-related HCC.

## MATERIALS AND METHODS

### Patients and samples

Between April 1998 and August 2006, 96 patients underwent surgical treatment of HCC at Yamaguchi University Hospital. All were positive for HCV antibody. Clinical characteristics of these patients based on the TNM classification (Sobin and Wittekind, 2002) of the Union Internationale Contre le Cancer (UICC) are shown in Table 1. To explore the relation between serum cfDNA levels and patient outcomes, we excluded six patients who had any residual tumour, and three patients who died of other disease. As a result, the remaining 87 patients were subjected to the following study. Patients were followed up as described previously (median follow-up time: 39 months) (Iizuka *et al*, 2003; Matoba *et al*, 2005). In brief, all 87 patients were followed up at least once every 3 months postoperatively by routine X-ray, ultrasonography (US), computed tomography, or magnetic resonance imaging, levels of serum *α*-fetoprotein and protein induced by vitamin K absence II (PIVKA-II) were also measured. When tumour recurrence was suspected, CT angiography was included as a follow-up examination.

For control, we used 100 serum samples from 100 HCV-positive patients with chronic liver disease who were recruited from outpatient clinics of the Yamaguchi University Graduate School of Medicine between July 2001 and October 2006. These control samples were selected to minimize the difference in age (66.3±7.3 *vs* 64.9±8.4 years (mean±s.d.), *P*=0.209) between the HCC group and the control group. Laboratory tests and imaging studies including US and CT did not reveal any HCC in the 100 HCV-positive patients during the median follow-up time of 18 months.

The study protocol was approved by the Institutional Review Board for the Use of Human Subjects at the Yamaguchi University School of Medicine, and written informed consent was obtained from each patient.

### Extraction and quantification of DNA in sera

Blood samples were collected as described previously (Iizuka *et al*, 2006a). After clotting, which occurred within 1 h of collection, blood samples were spun at 3000 r.p.m. (1600 × **g**) for 10 min at room temperature. Sera were stored at −80°C until use. DNA was extracted from 1 ml of serum with a DNA Extractor SP Kit for Serum and Plasma (Wako Pure Chemical Industries Ltd, Osaka, Japan) according to the manufacturer's instructions. DNA was quantified as described previously (Iizuka *et al*, 2006a). Briefly, 1 *μ*l of DNA solution was subjected to real-time PCR amplification for quantitative analysis of the *GSTP1* gene. Finally, we calculated the amount of DNA on the basis of standard DNAs (leucocyte genomic DNAs) at 16–2000 ng ml^−1^.

### Statistical analysis

Values are shown as mean±s.d. The Mann–Whitney *U*-test, Student's *t*-test and *χ*^2^ test were used to analyse differences in values between two groups, and analysis of variance was used to analyse differences between three groups. Overall survival and disease-free survival (DFS) were determined by the Kaplan–Meier method and analysed by log-rank test. The effect of eight clinicopathologic factors (sex, age, cfDNA level, tumour size, number of primary lesion, venous invasion, tumour differentiation grade and TNM stage) on OS and EHR in distant organs was assessed by means of the Cox proportional hazards model, hazard ratios (HRs) and 95% confidence intervals (CIs) were calculated. Multivariate analysis was also performed to identify independent factors for early IHR by means of the stepwise logistic regression model. The eight above-mentioned clinicopathologic factors were also entered into a forward stepwise regression model. Each model was tested for goodness of fit by –2 log likelihood and *χ*^2^ in each step. All analyses were performed with SPSS 11.0J software (SPSS Inc., Chicago, IL, USA) run on a Windows computer. A *P*-value of less than 0.05 was considered statistically significant.

## RESULTS

**Figure 1:**
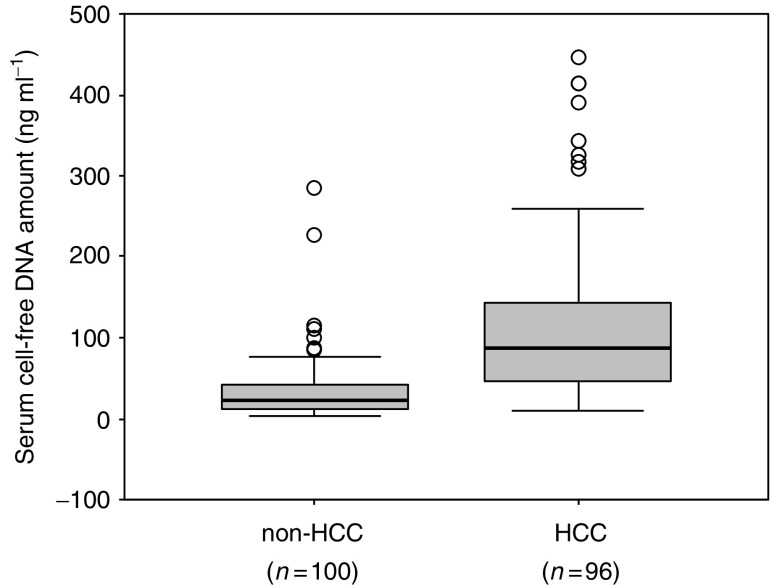
Box and whisker plot of cell-free DNA (cfDNA) levels in sera as determined by *GSTP1*-specific PCR assay. Levels of cfDNA in sera were significantly higher in hepatocellular carcinoma (HCC) patients than in hepatitis C virus (HCV) carriers (115.9±98.3 *vs* 34.4±40.4 ng ml^−1^ (mean±s.d.), *P*<0.0001 by Mann–Whitney *U*-test).

Serum cfDNA levels were significantly higher in HCC patients than in HCV carriers without known HCC (115.9±98.3 *vs* 34.4±40.4 ng ml^−1^ (mean±s.d.), *P*<0.0001 by Mann–Whitney *U*-test) (Figure 1). There was no significant difference in serum cfDNA levels between the 100 HCV carriers without known HCC and 18 healthy people or patients with benign disease who had no HCV infection (34.4±40.4 *vs* 45.8±22.6 ng ml^−1^); (data not shown). Serum cfDNA levels were not associated with any clinicopathologic factors in the total 96 HCC patients (Table 1).

**Figure 2:**
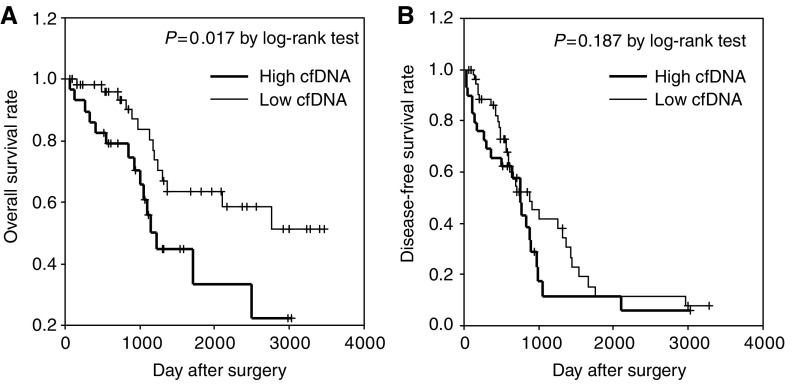
Relation between serum cell-free DNA (cfDNA) levels and overall and disease-free survival (DFS) in hepatocellular carcinoma (HCC) patients. (**A**) cfDNA levels and overall survival (OS). (**B**) cfDNA levels and DFS. Note that patients with a high cfDNA level had significantly shorter OS than did those with a low level of cfDNA (*n*=58) (*P*=0.017 by log-rank test). By contrast, serum cfDNA levels were not related to DFS.

Of the 87 patients who underwent curative hepatectomy, 35 had no recurrence during the follow-up period and the remaining 52 had IHR. Of the 52 patients with IHR, 13 had EHR in distant organs such as lung and bone. Given the finding that cfDNA levels of HCV carrier without HCC are similar to those of controls without HCV infection, to examine the relation between cfDNA and follow-up data, we used a cfDNA cutoff value of 117.8 ng ml^−1^, which is equal to the mean+2 s.d. of the control value (value in HCV carriers without HCC). Patients with a high cfDNA level (*n*=29) had significantly shorter OS than those with a low cfDNA level (*n*=58) (*P*=0.017 by log-rank test; Figure 2A). By contrast, serum cfDNA levels were not associated with DFS (Figure 2B). According to the multivariate Cox proportional hazards model, cfDNA (HR, 3.4; 95% CI, 1.5–7.6; *P*=0.004) and tumour size (HR, 3.8; 95% CI, 1.7–8.5; *P*=0.001) were the only independent prognostic factors for OS (Table 2). In addition, cfDNA (HR, 4.5; 95% CI, 1.3–14.9; *P*=0.014) was the only independent prognostic factor for EHR in distant organs (Table 3).

**Figure 3:**
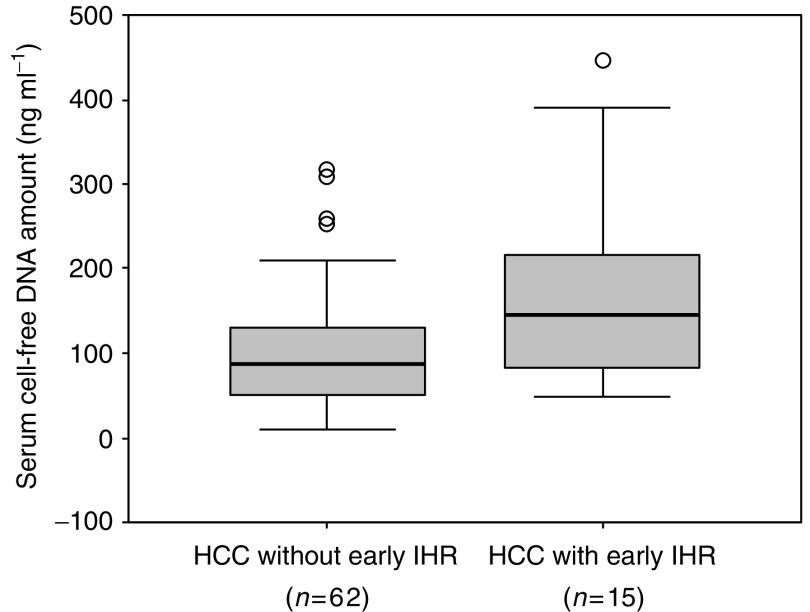
Box and whisker plot of cell-free DNA (cfDNA) levels in sera from patients with early intrahepatic recurrence (IHR) and patients without. Levels of cfDNA in sera were significantly higher in patients with early IHR than in patients without early IHR (176.3±124.8 *vs* 108.7±87.9 ng ml^−1^ (mean±s.d.), *P*=0.017 by Student's *t*-test).

To investigate the relation between cfDNA level and early IHR due to intrahepatic metastasis of HCC, we excluded 10 patients who had the follow-up periods of less than 1 year after surgery. Among the eligible 77 patients, 15 (19.5%) had early IHR within 1 year of surgery and the remaining 62 (80.5%) did not have early IHR. Among the 62 patients, 37 had IHR 1 year or more after surgery and 25 had no IHR during follow-up periods. The serum cfDNA level was significantly higher in patients (*n*=15) with early IHR than in those (*n*=62) without early IHR (176.3±124.8 *vs* 108.7±87.9 ng ml^−1^, *P*=0.017 by Student's *t*-test; Figure 3). The logistic regression model showed that tumour size (relative risk, 7.3; 95% CI, 1.9–27.7; *P*=0.004) and number of primary lesions (relative risk, 4.5; 95% CI, 1.1–17.7; *P*=0.033), but not cfDNA level, were independent risk factors for early IHR (data not shown).

## DISCUSSION

The possibility of detecting and measuring tumour-derived cfDNA has opened a new avenue in predictive oncology (Leon *et al*, 1977; Anker *et al*, 1999; Ziegler *et al*, 2002). This method provides a non-invasive and easy-to-use tool for screening for malignancy and predicting cancer outcomes. By means of real-time PCR, we showed previously that cfDNA levels were significantly higher in sera from patients with HCV-related HCC than in sera from HCV carriers without known HCC, and the diagnostic performance of cfDNA was superior to that of two representative HCC markers, *α*-fetoprotein and PIVKA-II (Iizuka *et al*, 2006a). Our present study confirmed the significantly increased levels of serum cfDNA in a large group of patients with HCV-related HCC, suggesting that the increase is related to progression of the disease caused by HCV infection. Indeed, our present study identified two subgroups of patients with HCV-related HCC: those with a high cfDNA level who had an unfavourable outcome after curative surgery, and those with a low cfDNA level, who had a favourable outcome. Notably, cfDNA was the only independent prognostic factor for EHR in distant organs in case of HCV-related HCC treated surgically.

Recurrence of HCC is quite complicated. There are at least three representative modes of postoperative recurrence, early and late IHRs appearing in the remnant liver and EHR appearing in distant organs such as bone and lung. Among the three types of recurrence, late IHR is a *de novo* primary tumour rather than a metastatic tumour, and it accounts for the majority of HCC recurrences 3 years or more after surgery (Kumada *et al*, 1997), suggesting that it has less influence on patient survival. The finding that our cohort included many patients with late IHR might account for the lack of association between cfDNA levels and DFS in patients with HCV-related HCC. In contrast, our univariate analysis showed increased levels of cfDNA in HCC patients with early IHR. Most early IHRs can be attributed to intrahepatic metastasis of cancer cells and are detected in 30–50% of patients within 1 or 2 years after surgery, limiting the potential for surgical cure of HCC (Tung-Ping Poon *et al*, 2000; Iizuka *et al*, 2003; Llovet *et al*, 2003; Matoba *et al*, 2005; Portolani *et al*, 2006). Thus, the increased levels of serum cfDNA are related to the high metastatic potential, but not to the appearance of *de novo* tumour, of this type HCC.

Much effort has been devoted to developing predictive makers for early IHR. Some recent molecular profiling studies (Iizuka *et al*, 2003, 2004; Kurokawa *et al*, 2004) raised the possibility of accurately predicting early IHR in a cohort of patients with HCC, most of which were HCV-related HCCs. Predictive systems that are developed will enable to accurately detect patients at high risk for early IHR, but there might be many problems to solve before these markers can be applied to daily clinical practice (Iizuka *et al*, 2004). Thus far, there have been few reports on a predictor for EHR after surgery, although several studies have identified several key genes or gene products related to distant metastasis (Schimanski *et al*, 2006; Iizuka *et al*, 2006b). It was reported that HCC recurred in distant organs in only 3 (7%) of 42 patients who underwent liver transplantation, a radical curative treatment strategies (Nart *et al*, 2003). A recent large study by Yang *et al* (2007) showed that, among 348 HCC patients who underwent hepatectomy, 47 (13.5%) had EHR during the follow-up period of 4.8±3.7 years. Thus, the frequency of EHR is lower than that of early IHR; however, once HCC progresses to EHR, it is difficult to control the lesions in most cases because of the limited resectability. Indeed, the frequency of death due to respiratory failure resulting from metastasis of HCC to the lung has increased over the last 30 years in the Japanese population (Itoh *et al*, 2002). In this regard, our present finding that a patient with a high level of cfDNA has 4.5-fold increased risk for EHR in distant organs may be clinically useful. Such a robust predictive system is urgently needed to screen for patients who will develop EHR.

Taken together, our data suggest that serum cfDNA levels could serve as a useful tool for prediction of EHR after curative surgery in patients with HCV-related HCC. However, it is unlikely that cfDNA alone will be effective in predicting EHR in more global cases. A large training-validation study is needed to construct a robust predictor made up of multiple factors in which cfDNA might play a central role. In addition, identifying and quantifying genetic changes in circulating cfDNAs specific to HCV-related HCC will allow us to determine metastatic potential preoperatively on an individual basis.

## Figures and Tables

**Table 1:** Characteristics of patients and HCCs per cfDNA level

	**cfDNA amount**		
	**Low (<117.8 ng ml^−1^)**	**High (⩾117.8 ng ml^−1^)**	***P*-value**
*Sex*			0.132
Male (*n*=71)	42	29	
Female (*n*=25)	19	6	
			
*Age* (*year*)			0.76
<60 (*n*=18)	12	6	
>60 (*n*=78)	49	29	
			
*Tumour size*			0.879
<5 cm (*n*=76)	48	28	
>5 cm (*n*=20)	13	7	
			
*Number of primary lesion*			0.299
Single (*n*=56)	38	18	
Multiple (*n*=40)	23	17	
			
*Venous invasion*			0.586
Negative (*n*=69)	45	24	
Positive (*n*=27)	16	11	
			
*Tumour differentiation*			0.155
Well (G1) (*n*=24)	18	6	
Moderately (G2) (*n*=64)	40	24	
Poorly (G3) (*n*=8)	3	5	
			
*UICC TNM stage*			0.217
I (*n*=44)	32	12	
II (*n*=42)	23	19	
IIIA/IV (*n*=10)	6	4	

cfDNA=cell-free DNA; HCV=hepatitis C virus; HCC=hepatocellular carcinoma.

To evaluate the high and low of cfDNA levels, we used a cutoff value of 117.8 ng ml^−1^, which is equal to the mean+2s.d. of the control value (value in HCV carriers without HCC).

**Table 2:** Independent risk factors for OS

**Variable**	**HR (95% CI)**	***P*-value**
*cfDNA*		0.004
Low^a^	1	
High^a^	3.4 (1.5–7.6)	
		
*Tumour size*		0.001
Less than 5 cm	1	
More than or equal to 5 cm	3.8 (1.7–8.5)	

cfDNA=cell-free DNA; CI=confidence interval; HCC=hepatocellular carcinoma; HR=hazard ratio; OS=overall survival.

a Low, less than 117.8 ng ml^−1^; high, more than or equal to 117.8 ng ml^−1^.

**Table 3:** Independent risk factors for cancer recurrence in distant organs

**Variable**	**HR (95% CI)**	***P*-value**
*cfDNA*		0.014
Low^a^	1	
High^a^	4.5 (1.3–14.9)	
		
*Tumour differentiation grade*		0.069
G1^b^	1	
G2+G3^b^	2.5 (0.9–7.0)	

cfDNA=cell-free DNA; CI=confidence interval; HCC=hepatocellular carcinoma; HR=hazard ratio.

a Low, less than 117.8 ng ml^−1^; high, more than or equal to 117.8 ng ml^−1^.

b G1, well-differentiated HCC; G2, moderately differentiated HCC; G3, poorly differentiated HCC.

## References

[bib1] Anker P, Mulcahy H, Chen XQ, Stroun M (1999) Detection of circulating tumour DNA in the blood (plasma/serum) of cancer patients. Cancer Metastasis Rev 18: 65–73 10505546 10.1023/a:1006260319913

[bib2] Deuffic S, Poynard T, Buffat L, Valleron AJ (1998) Trends in primary liver cancer. Lancet 351: 214–215 10.1016/S0140-6736(05)78179-4 9449893

[bib3] El Serag HB (2004) Hepatocellular carcinoma: recent trends in the United States. Gastroenterology 127: S27–S34 15508094 10.1053/j.gastro.2004.09.013

[bib4] Gautschi O, Bigosch C, Huegli B, Jermann M, Marx A, Chasse E, Ratschiller D, Weder W, Joerger M, Betticher DC, Stahel RA, Ziegler A (2004) Circulating deoxyribonucleic acid as prognostic marker in non-small-cell lung cancer patients undergoing chemotherapy. J Clin Oncol 22: 4157–4164 15483026 10.1200/JCO.2004.11.123

[bib5] Iizuka N, Hamamoto Y, Oka M (2004) Predicting individual outcomes in hepatocellular carcinoma. Lancet 364: 1837–1839 15555651 10.1016/S0140-6736(04)17455-2

[bib6] Iizuka N, Oka M, Okabe H, Nishida M, Maeda Y, Mori N, Takao T, Tamesa T, Tangoku A, Tabuchi H, Hamada K, Nakayama H, Ishitsuka H, Miyamoto T, Hirabayashi A, Uchimura S, Hamamoto Y (2003) Oligonucleotide microarray for prediction of early intrahepatic recurrence of hepatocellular carcinoma after curative resection. Lancet 361: 923–929 12648972 10.1016/S0140-6736(03)12775-4

[bib7] Iizuka N, Oka M, Yamada-Okabe H, Mori N, Tamesa T, Okada T, Takemoto N, Tangoku T, Hamada K, Nakayama H, Miyamoto T, Uchimura S, Hamamoto Y (2002) Comparison of gene expression profiles between hepatitis B virus- and hepatitis C virus-infected hepatocellular carcinoma by oligonucleotide microarray data on the basis of a supervised learning method. Cancer Res 62: 3939–3944 12124323

[bib8] Iizuka N, Sakaida I, Moribe T, Fujita N, Miura T, Stark M, Tamatsukuri S, Ishitsuka H, Uchida K, Terai S, Sakamoto K, Tamesa T, Oka M (2006a) Elevated levels of circulating cell-free DNA in the blood of patients with hepatitis C virus-associated hepatocellular carcinoma. Anticancer Res 26: 4713–4719 17214331

[bib9] Iizuka N, Tamesa T, Sakamoto K, Miyamoto T, Hamamoto Y, Oka M (2006b) Different molecular pathways determining extrahepatic and intrahepatic recurrences of hepatocellular carcinoma. Oncol Rep 16: 1137–1142 17016605

[bib10] Itoh Y, Ohkubo K, Iuchi H, Michitaka K, Horiike N, Onji M (2002) Chronological changes of causes of death and distant metastasis in hepatocellular carcinoma. Oncol Rep 9: 331–335 11836602

[bib11] Kumada T, Nakano S, Takeda I, Sugiyama K, Osada T, Kiriyama S, Sone Y, Toyoda H, Shimada S, Takahashi M, Sassa T (1997) Patterns of recurrence after initial treatment in patients with small hepatocellular carcinoma. Hepatology 25: 87–92 8985270 10.1053/jhep.1997.v25.pm0008985270

[bib12] Kuramitsu Y, Nakamura K (2006) Proteomic analysis of cancer tissues: shedding light on carcinogenesis and possible biomarkers. Proteomics 6: 5650–5661 16972299 10.1002/pmic.200600218

[bib13] Kurokawa Y, Matoba R, Takemasa I, Nagano H, Dono K, Nakamori S, Umeshita K, Sakon M, Ueno N, Oba S, Ishii S, Kato K, Monden M (2004) Molecular-based prediction of early recurrence in hepatocellular carcinoma. J Hepatol 41: 284–291 15288478 10.1016/j.jhep.2004.04.031

[bib14] Leon SA, Shapiro B, Sklaroff DM, Yaros MJ (1977) Free DNA in the serum of cancer patients and the effect of therapy. Cancer Res 37: 646–650 837366

[bib15] Llovet JM, Burroughs A, Bruix J (2003) Hepatocellular carcinoma. Lancet 362: 1907–1917 14667750 10.1016/S0140-6736(03)14964-1

[bib16] Mann CD, Neal CP, Garcea G, Manson MM, Dennison AR, Berry DP (2007) Prognostic molecular markers in hepatocellular carcinoma: a systematic review. Eur J Cancer 43: 979–992 17291746 10.1016/j.ejca.2007.01.004

[bib17] Marrero JA, Lok AS (2004) Newer markers for hepatocellular carcinoma. Gastroenterology 127: S113–S119 15508074 10.1053/j.gastro.2004.09.024

[bib18] Matoba K, Iizuka N, Gondo T, Ishihara T, Yamada-Okabe H, Tamesa T, Takemoto N, Hashimoto K, Sakamoto K, Miyamoto T, Uchimura S, Hamamoto Y, Oka M (2005) Tumor HLA-DR expression linked to early intrahepatic recurrence of hepatocellular carcinoma. Int J Cancer 115: 231–240 15688398 10.1002/ijc.20860

[bib19] Nart D, Arikan C, Akyildiz M, Yuce G, Demirpolat G, Zeytunlu M, Karasu Z, Aydogdu S, Killi R, Yuzer Y, Tokat Y, Kilic M (2003) Hepatocellular carcinoma in liver transplant era: a clinicopathologic analysis. Transplant Proc 35: 2986–2990 14697957 10.1016/j.transproceed.2003.10.076

[bib20] Parkin DM, Bray F, Ferlay J, Pisani P (2001) Estimating the world cancer burden: Globocan 2000. Int J Cancer 94: 153–156 11668491 10.1002/ijc.1440

[bib21] Portolani N, Coniglio A, Ghidoni S, Giovanelli M, Benetti A, Tiberio GA, Giulini SM (2006) Early and late recurrence after liver resection for hepatocellular carcinoma: prognostic and therapeutic implications. Ann Surg 243: 229–235 16432356 10.1097/01.sla.0000197706.21803.a1 PMC1448919

[bib22] Ren N, Qin LX, Tu H, Liu YK, Zhang BH, Tang ZY (2006) The prognostic value of circulating plasma DNA level and its allelic imbalance on chromosome 8p in patients with hepatocellular carcinoma. J Cancer Res Clin Oncol 132: 399–407 16502316 10.1007/s00432-005-0049-5 PMC12161085

[bib23] Schimanski CC, Bahre R, Gockel I, Muller A, Frerichs K, Horner V, Teufel A, Simiantonaki N, Biesterfeld S, Wehler T, Schuler M, Achenbach T, Junginger T, Galle PR, Moehler M (2006) Dissemination of hepatocellular carcinoma is mediated via chemokine receptor CXCR4. Br J Cancer 95: 210–217 16819541 10.1038/sj.bjc.6603251 PMC2360625

[bib24] Sobin LH, Wittekind C (2002) TNM classification of Malignant Tumours, 6th edn. UICC: Wiley-Liss. 81–83

[bib25] Tung-Ping Poon R, Fan ST, Wong J (2000) Risk factors, prevention, and management of postoperative recurrence after resection of hepatocellular carcinoma. Ann Surg 232: 10–24 10862190 10.1097/00000658-200007000-00003 PMC1421103

[bib26] Umetani N, Giuliano AE, Hiramatsu SH, Amersi F, Nakagawa T, Martino S, Hoon DS (2006) Prediction of breast tumor progression by integrity of free circulating DNA in serum. J Clin Oncol 24: 4270–4276 16963729 10.1200/JCO.2006.05.9493

[bib27] Yang Y, Nagano H, Ota H, Morimoto O, Nakamura M, Wada H, Noda T, Damdinsuren B, Marubashi S, Miyamoto A, Takeda Y, Dono K, Umeshita K, Nakamori S, Wakasa K, Sakon M, Monden M (2007) Patterns and clinicopathologic features of extrahepatic recurrence of hepatocellular carcinoma after curative resection. Surgery 141: 196–202 17263976 10.1016/j.surg.2006.06.033

[bib28] Ziegler A, Zangemeister-Wittke U, Stahel RA (2002) Circulating DNA: a new diagnostic gold mine? Cancer Treat Rev 28: 255–271 12435372 10.1016/s0305-7372(02)00077-4

